# Supplementary material for: ProteinShader: illustrative rendering of macromolecules
Source: BMC Struct Biol. 2009 Mar 30;9:19. doi: 10.1186/1472-6807-9-19 (PMC2672931; doi:10.1186/1472-6807-9-19)
Supplement: Additional file 1 — ProteinShader program without source code. This compressed file contains the complete ProteinShader program including associated libraries, but no source code. A README.txt file gives an overview of the ProteinShader distribution, and the index.html file in the help subdirectory has directions on getting started with the program as well as a set of tutorials. [file 1472-6807-9-19-S1.zip › ProteinShader-beta-0_9_4-binary/help/api/org/proteinshader/structure/visitor/modifiers/package-summary.html]

org.proteinshader.structure.visitor.modifiers (ProteinShader API)


|  |  |  |
| --- | --- | --- |
| **Overview** \| **Package** \| Class \| **Use** \| **Tree** \| **Deprecated** \| **Index** \| **Help** | |  |
| **PREV PACKAGE**   NEXT PACKAGE | **FRAMES**    **NO FRAMES**     **All Classes** |  |


---

## Package org.proteinshader.structure.visitor.modifiers

Holds helper classes for the ModifierVisitor class, which is used to modify
Drawable objects (Atoms, Bonds, and Segments).

**See:**
  
          **Description**

| **Class Summary** | |
| --- | --- |
| **AtomModifier** | This helper class for the ModifierVisitor knows how to accept an Atom object and modify its attributes. |
| **BondModifier** | This helper class for the ModifierVisitor knows how to accept a Bond object and modify its attributes. |
| **DrawableModifier** | This helper class for the ModifierVisitor knows how to accept a Drawable object and modify its attributes. |
| **SegmentModifier** | This helper class for the ModifierVisitor knows how to accept a Segment object and modify its attributes. |

## Package org.proteinshader.structure.visitor.modifiers Description

Holds helper classes for the ModifierVisitor class, which is used to modify
Drawable objects (Atoms, Bonds, and Segments).
